# Professional Gossip

**Published:** 1848-07

**Authors:** 


### PROFESSIONAL GOSSIP.

We confess that the shifts and turns resorted to by various members of the Profession to secure patronage, *so called*, often strike us as exceedingly repulsive, and, in onr view, very much lowers the standard Dentistry should assume and maintain as a useful and liberal profession. We design not to specially notice the *dental pendlar*, who, with kit in hand, beats up the lanes and avenues of our city—sometimes forcing his way into the mansions of ease and plenty; our gossip is not, for the present, with such; not, however, for the want of material to fill up a page; of such, we could *cull* items for a small pocket manuel at least. But we shall take the other extreme, and give as we heard it, what appeared to us a rich and racy scene in the bold maneuvering for honor, place, and fortune, and admirably shows the manner in which names are sometimes secured as reference, and also shows that sterling worth which knows, and can act from principle.

But to our gossip. In one of our western states, not over one thonsand miles from this, flourishes a pompous, consequential, and withal, a tolerable good Dentist, but fond of desplay, and loves the uppermost seat in the synagogue.

Business took him to the capital of the state, during the sitting of the Legislature; and hearing that senator T's lady needed a full set of teeth, he immedlately called on the Hon. gentleman and introduced himself as Dr. W., adding an M. and several D.'s to his name, and solicited the favor of inserting the teeth for his lady.

The Hon. gentleman, thinking one so well armed with degrees, should be capable, consented that he might go to his residence, some forty miles distant, and, if his lady seen proper, might operate. This was accomplished, and, we presume, the teeth inserted to the satisfaction of the lady; for, on his return, senator T. consented to the use of his name as reference; so, also, the secretary of state, and perhaps others of note; still, however, the Governor's name and influence was not secured, and a card thus deficient would be of but little service in his estimation. This must then be obtained. Words will here fail to depict the modus operandi for its accomplishment. Believing that "a faint heart would never win a fair lady," he thought also that a bold front would most likely succeed with the Governor. Immagine, then, the senate in session—an important and interesting discussion going on. The Gov. had dropped in to listen, and was deeply attentive to the arguments pro. and con. being delivered. The Dr. enters the hall—clears his throat—gives an emphatic hem, the clear shrill tones of which startles the house—the Gov. turns his head—the Dr. beckons—all is silent—the member resumes his speech—the Gov. turns to listen—another hem and cough from the Dr.—another pause—the Dr. beckons the Gov. again. Thus, after three or four repetitions, the worthv Gov. determines to relieve the suspense of the house and see what the stranger wanted. They pass out, and the Dr., with all his suavity of manner, approaching the chief magistate of a great state, remarks, Governor B., I am Dr. W., adding, of course, the M. and D.'s, and purpose remaining a short time at the seat of government. I have inserted a full set of teeth for senator T.'s lady—have his signature, the secretary of state's, and others to my card, and would be glad to have your honor's name at the head of the list. The science of dentistry, Governor, is a noble science. The good people of your city have, I know, been miserably humbugged. I wish to render them essential service, and wipe off that stain from our profession, which injures so much the cause of true science, and show the inhabitants of this place that every Dentist is not a quack. Well, Dr. at length puts in the Governor, I *don't know you.* What! not know ME, Gov.; have'nt you heard of my great reputation? No, I don't know you, Dr.; yet if senator T. says you are a good Dentist, I can certify to his veracity—I can recommend *him.* Here the first scene of this *mellow drama* ends.

Scene 2d. The boarding house of the Governor—our hero, without his (the Gov.'s) knowledge, wishing to be at the head of the pot—had taken rooms at the same hotel—dinner is announced—a crowd passes to the table. Gov. B., meeting a Dr. of his acquaintance, speaks out in not a very low voice, Dr., who is this Dr. W., Dentist, &c., &c., who figures so largely in the dental way? A few knowing nods and significant jestures from his friend, soon gave the Governor to understand that the object of his inquiry was at hand. The curtain here drops, just as the Dr. slopes from the dinner table.—CIN. ED.

---

GOSSIP has also supplied us with the following: A very nervous lady, getting a tooth extracted in one of our dental offices of this city, a few days since, as soon as the tooth was extracted, throws herself on the floor, and in her pain and fright, rolls on the rug, with her head on the fender, exclaiming she was killed—her head was torn off! Oh! I am dead, &c., &c. Our worthy friend, to divert her attention, approached her, remarking very patheticly, that he would not mind all that, if her leg was not broken. Och! Dr. and is my leg broken, she exclaims, and up she jums to ascertain the fact. By this time she concluded she was not quite dead, and that her head was still on her shoulders.—CIN. ED.

